# CD10 Immunohistochemical Expression in Breast Carcinoma and Its Correlation With Clinicopathological Parameters

**DOI:** 10.7759/cureus.67279

**Published:** 2024-08-20

**Authors:** Vijayalaxmi S Patil, Shraddha Barate

**Affiliations:** 1 Pathology, Shri B. M. Patil Medical College, Hospital and Research Centre, Bijapur Lingayat District Education (Deemed to Be University), Vijayapura, IND; 2 Pathology, Lilawati Heart Care and Medical Hospital, Lunawada, IND

**Keywords:** tumor grade, er/pr, prognosis, immunohistochemistry, stromal cd10, breast cancer

## Abstract

Background: Interaction between the stromal and tumor cells is of crucial importance in breast cancer progression and response to therapy. A literature search has shown that stromal CD10 expression signifies the biological aggressiveness of various epithelial malignancies. Stromal markers are now becoming apparent as novel markers in evaluating the prognosis of invasive breast cancer and have not been studied substantially to date.

Objectives: To study the immunohistochemical expression of CD10 in stromal cells of breast carcinoma and to correlate the expression of CD10 with various clinicopathological prognostic factors such as the size of the tumor, histological grade, lymph node status, and estrogen receptor (ER), progesterone receptor (PR), and human epidermal growth factor receptor 2/neu protooncogene (HER2-neu) status.

Methodology: In the present study, a hospital-based cross-sectional study was conducted on 50 mastectomy specimens diagnosed with invasive breast carcinoma. The specimens of patients who had received neoadjuvant therapy or chemotherapy were excluded. Size of the tumor, grade of tumor on histopathology, lymph node involvement, and IHC status of ER, PR, and HER2-neu were noted. IHC staining for the CD10 marker was performed, and expression of stromal CD10 was correlated with these clinical-pathological prognostic factors.

Results: CD10 expression in stromal cells of breast carcinoma was seen in 40 (80%) cases, and it showed a statistically significant association with histological grade (χ^2^ = 17.262; p-value < 0.0001), ER negativity (χ^2^ = 3.668; p-value < 0.045), and PR negativity (χ^2^ = 3.926; p-value < 0.048).

Conclusion: A strong association of stromal CD10 expression with a well-established negative prognostic marker such as a higher tumor grade, ER-negative status, and PR-negative status was noted and thus, stromal CD10 expression can be used as an independent prognostic marker in breast carcinoma.

## Introduction

Breast cancer poses a serious threat to women's lives and health, and it has drawn attention from a number of sectors. Data from the 2018 Global Cancer Survey show that among women worldwide, carcinoma breast has the highest incidence of malignant tumors, accounts for 15% of all malignant tumor-related mortality, ranks sixth in the death rate of malignant tumors, and exhibits a tendency for rapid increase [1]. Breast cancer constitutes 18.5% of all new cancer diagnoses in India [2].

The development, progression, and prognosis of cancer are all closely related to the tumor immune microenvironment, which also serves as the site of immunological escape and tumor cell immune surveillance [3]. Breast cancer exhibits heterogeneity in its histopathological characteristics, metastatic patterns, molecular features, outcomes, and therapeutic responses. This variability is driven by the interactions between tumor cells and the surrounding microenvironment [4].

Well-acknowledged conventional clinicopathological prognostic factors like histological tumor grade, lymph node metastases, human epidermal growth factor receptor 2/neu protooncogene (HER2-neu), progesterone receptor (PR), and estrogen receptor (ER) status are routinely considered in all probable cases of carcinoma of the breast. Stroma has an important function in modifying and regulating tumor infiltration and metastasis [5].

It is known that CD10 expression in tumor stroma is closely associated with higher tumor grade/tumor stage and thus signifies the biological aggressiveness of various malignancies belonging to the lining epithelium [6]. Since they have not been extensively researched up to this point, stromal markers are gradually becoming more prevalent as new indicators for determining carcinoma breast prognosis [7].

The purpose of the present study was to analyze the immunohistochemical CD10 expression in the stromal cells in invasive breast cancer and the association of the CD10 expression with several prognostic markers such as the age of the patient, size of the tumor, lymph node status, histological grade, stage of the tumor, and HER2-neu, PR, and ER status.

## Materials and methods

**Figure 1:**
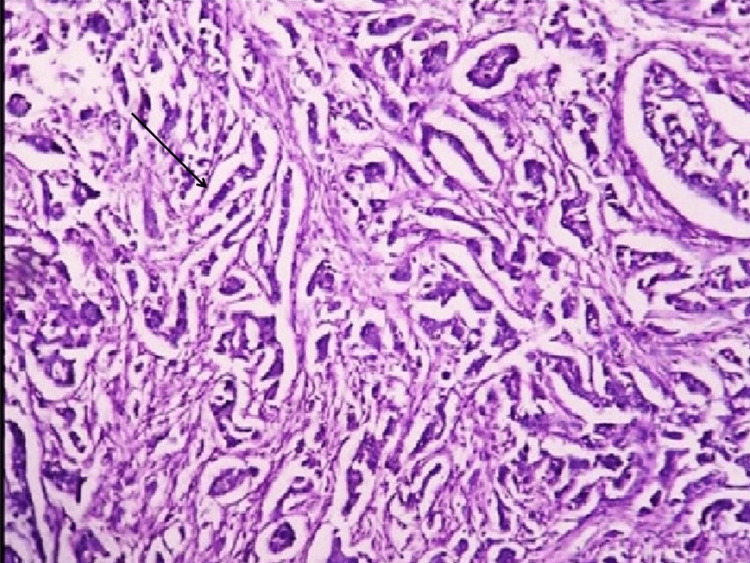
Photomicrograph of invasive breast carcinoma with an arrow showing infiltrating cords of tumor cells (Hematoxylin and Eosin stain, 100x)

This cross-sectional study was carried out in the Histopathology Section of the Pathology Department at Shri B. M. Patil Medical College, Hospital and Research Centre, Bijapur Lingayat District Education (Deemed to Be University), Vijayapura, India. The ethical approval for the study was obtained from the Institutional Ethical Committee of Shri B. M. Patil Medical College, Hospital and Research Centre, Bijapur Lingayat District Education (Deemed to Be University) (IEC/No-09/2021). Fifty cases of invasive breast carcinoma, operated on from January 2020 to December 2022, were studied. Lumpectomy specimens and biopsy specimens were excluded from the study. The mastectomy specimens were examined grossly and preserved in 10% neutral buffered formalin; representative sections from the specimen were given and processed routinely, and slides were prepared and stained. Five four-micron thick sections were prepared from the most suitable tumor tissue block. Hematoxylin and Eosin (H&E) staining (Figure 1) was used on one tissue section to make a morphologic diagnosis and Modified Bloom‑Richardson system of cancer grading.

Immunohistochemical staining

Four µm sections were mounted on poly L lysine-coated slides, deparaffinized, and rehydrated, and antigen retrieval was performed with Tris buffer. After the peroxide block, the sections were incubated with primary antibodies (ER, PR, HER2-neu, CD10 monoclonal mouse antihuman antibody, clone 56C6, Dako) for 30 minutes, washed with wash buffer, and further incubated with labeled polymer horseradish peroxidase (HRP) (DakoEn Vision + Dual Link System, HRP, DAB +, Code K4065). The bound antibodies were visualized using a DAB (3,3'-diaminobenzidine) chromogen, counterstained with hematoxylin, and mounted. The immunohistochemical (nuclear staining) expression of the markers ER and PR was evaluated based on the Allred scoring system, while HER2 was evaluated based on the extent and intensity of membrane staining. In the evaluation of CD10 expression, when more than 10% of stromal cells showed cytoplasmic positivity, they were interpreted as CD10-positive [8]. The details of the patient, including the age, size of the tumor, grade of tumor on histopathology, and lymph node involvement, were obtained. The expression of CD10 was correlated with clinicopathological variables such as age, tumor size, histological grade, metastasis to lymph nodes, HER2-neu, PR, and ER status.

The data were entered into a Microsoft Excel sheet (Microsoft Corp., Redmond, United States) and statistical analysis was performed using a statistical package for the social sciences (IBM SPSS Statistics for Windows, Version 20 (Released 2011; IBM Corp., Armonk, New York, United States)). Quantitative data were presented with the help of mean and qualitative data were presented with the help of percentages and diagrams. Association between the variables was found using the chi-square test. A p-value of less than 0.05 was considered statistically significant.

## Results

**Figure 2:**
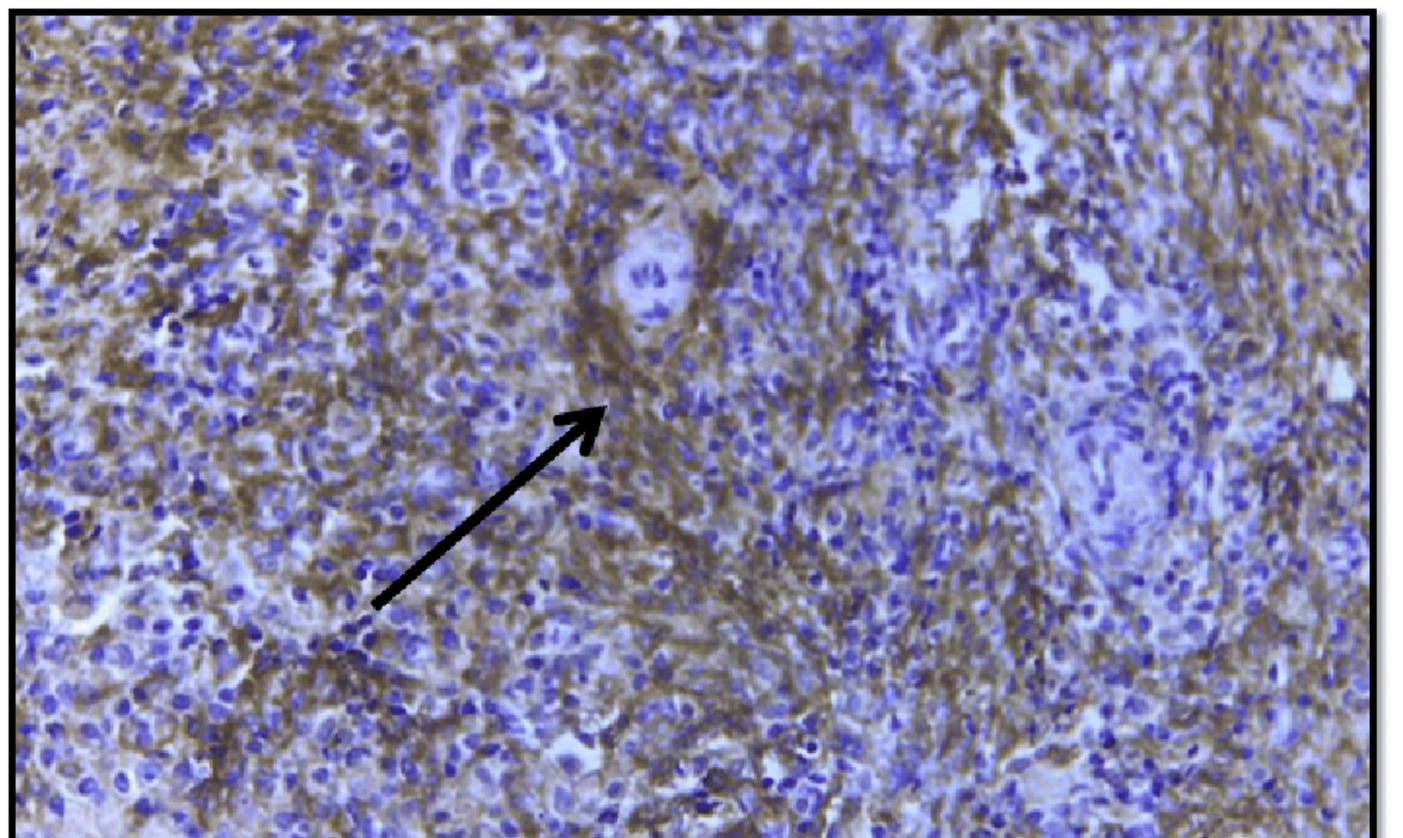
Photomicrograph of invasive breast carcinoma with an arrow showing CD10 cytoplasmic expression in stromal cells (400x)

We studied 50 cases of invasive breast carcinoma. The mean age of the patients in this study was 52 years, with a range of 30 to 80 years. Among the 50 cases included in the study sample, 40 (80%) cases demonstrated CD10 expression in tumor stromal cells on immunohistochemistry (Figure 2). There were five cases in the T1 stage among which four cases (80%) showed stromal CD10 expression, 32 cases in T2 among which 26 cases (81.2%) showed stromal CD10 expression, and 11 cases of T3 with eight cases (72.7%) among them showing stromal CD10 expression. ER-positive expression was noted in 40 cases among which 23 (57.5%) cases showed stromal CD10 expression while nine cases (90%) out of 10 ER-negative cases showed stromal CD10 expression. PR-positive expression was noted in 40 cases among which 18 (45%) cases showed stromal CD10 expression while eight cases (80%) out of 10 PR-negative cases showed stromal CD10 expression. Among the 50 cases studied, HER2 expression was noted in 40 cases, among which 17 cases (42.5%) showed CD10 expression, and among 10 cases of negative expression, seven cases (70%) showed CD10 expression.

Expression of CD10 by the stromal cells in cases of carcinoma breast and its correlation with clinicopathological parameters are shown in Table 1.

**Table 1:** Association of stromal CD10 expression with clinicopathological prognostic factors ER: estrogen receptor; PR: progesterone receptor; HER2: human epidermal growth factor receptor ^*^: statistically significant (p < 0.05)

Parameters	Stromal CD10 expression		χ^2 ^(chi-square value)	p-value (<0.05 considered significant)
	No. of cases showing CD10 negativity (%)	No. of cases showing CD10 positivity (%)		
Age				
<50 years	5 (18.5%)	22 (81.5%)	0.081	0.777
>50 years	5 (21.7%)	18 (78.3%)		
Lymph node status				
Negative	5 (29.4%)	12 (70.6%)	1.462	0.232
Positive	5 (15.2%)	28 (84.8%)		
Tumor size				
T1	1 (20%)	4 (80%)	0.895	0.827
T2	6 (18.8%)	26 (81.2%)		
T3	3 (27.3%)	8 (72.7%)		
T4	0 (0%)	2 (100%)		
Histologic grade				
I	10 (47.6%)	11 (52.4%)	17.262	0.0001*
II	0 (0%)	23 (100%)		
III	0 (0%)	06 (100%)		
ER status				
Negative	1 (10%)	9 (90%)	3.668	0.045
Positive	17 (42.5%)	23 (57.5%)		
PR status				
Negative	2 (20%)	8 (80%)	3.926	0.048
Positive	22 (55%)	18 (45%)		
HER2 status				
Negative	3 (30%)	7 (70%)	2.242	0.119
Positive	23 (57.5%)	17 (42.5%)		

In this current study of 50 cases, the majority i.e., 23 (57.5%) cases, belonged to histologic grade II. Metastasis to lymph nodes was seen in 33 (66%) cases and the majority of the cases, i.e., 32 cases (64%) had a tumor size of 2-5 cm with ranges of the size of the tumor varying from 1.5 cm to 10 cm. We also assessed the ER, PR, and HER2-neu immunohistochemical status of the tumor and observed that among 10 cases with ER-negativity, nine cases (90%) showed stromal CD10 expression, 8 (80%) out of 10 PR negative cases showed stromal CD10 expression. Among the 40 cases with HER2 positivity, stromal CD10 expression was noted in 17 (42.5%) cases, and no expression was seen in 23 (57.5%) cases.

A positive correlation between stromal CD10 expression and histological grade was observed, which was significant statistically (χ^2^ = 17.262; p-value < 0.0001). Stromal positivity for CD10 expression showed a highly statistically significant association with ER negativity (χ^2^ = 3.668; p-value < 0.045) and PR negativity (χ^2^ = 3.926; p-value < 0.048). There was no positive association between stromal CD10 and the age of the patient, lymph node metastasis, tumor size, and HER2-neu status.

## Discussion

The matrix is crucial in cancer development, as matrix molecules modulate tumor invasion and metastasis. Gaining a better understanding of stromal contributions to cancer progression may help identify specific signals that promote growth, de-differentiation, invasion, and ectopic survival, ultimately leading to the discovery of new therapeutic targets and prospective indicators for future treatments [8-10].

The mutual efforts of epithelial cells and stromal components are responsible for maintaining the structural and functional integrity of the breast. Various factors are responsible for the interaction between tumor cells and stromal cells that regulate the growth of the tumor, adhesion, and migration, thereby affecting the invasiveness and metastatic potential of cancer cells. This modifying effect of matrix molecules grants the matrix a crucial role in cancer proliferation and metastasis [11].

Increasing our understanding of the role of stroma in breast cancer development and progression will aid in identifying new prognostic markers that can serve as potential therapeutic targets [7,12]. CD10, also known as "Common Acute Lymphoblastic Antigen," is a "zinc-dependent metalloproteinase" present on the surface of the cell, commonly expressed in lymphoid stem cells, bone marrow, mature neutrophils, pro-B lymphoblasts, various subtypes of lymphomas, endometrial stromal sarcomas, and renal cell carcinomas. Stromal CD10 expression has been linked to biological aggressiveness in a range of epithelial malignancies and phyllode tumors [6].

Normally in breast tissue, myoepithelial cells, lining the outer layer of the ducts, solely express CD10. However, in invasive breast carcinoma, there is an accumulation of CD10-cleaved peptides as the CD10 enzymatic activity is upregulated. These peptides inhibit epithelial cell differentiation, which promotes epithelial-mesenchymal transition and malignant proliferation [3,7]. We examined CD10 expression in stromal cells in the current investigation to determine whether CD10 is connected to a specific clinicopathological feature of breast cancer.

Our study included 50 cases of invasive breast carcinoma, among whom 80% of cases showed CD10 stromal positivity. A minimal to moderate deviation in CD10 expression was noted in a few studies, with some of the studies reporting CD10 expression up to 80% (Table 2).

**Table 2:** Comparison of stromal CD10 expression in the present study with other studies

Author	Sample size	No. of CD10 positive cases	Percentage of CD10 positive cases (%)
Present study	50	40	80%
Gaffoor et al. [2]	62	51	82.3%
Puri et al. [5]	50	40	80%
Dhande et al. [6]	60	47	78.3%
Jana et al. [7]	70	34	48.6%
Makretsov et al. [8]	258	204	79%
Iwaya et al. [13]	110	20	18%
Taghizadeh‑Kermani et al. [14]	100	64	64%
Kim et al. [15]	101	50	49.5%

There was no significant association of stromal CD10 positivity with the age of the patient. This finding was similar to the findings of studies conducted by Puri et al. [5], Dhande et al. [6], and Jana et al. [7].

On comparing the expression of CD10 in stromal cells with the histological grade of tumor, 11 (27.5%) cases out of 21 cases of grade I carcinoma showed CD10 positivity, while all the cases of grade II and grade III carcinoma showed CD10 positivity. Thus, the CD10 expression in stromal cells was higher with an increasing histological grade, suggesting a correlation between CD10 expression and higher tumor grade, which was statistically significant. This finding was consistent with results of the other studies by other researchers [2,5,6,8,12-14].

In our study, 26 cases of T2, eight cases of T3, and two cases of T4 showed CD10 expression with a p-value of 0.827, showing no significant association of stromal expression of CD10 with the size of the tumor. This result was similar to the results of other studies [5-8]. In the study, we did not find a significant correlation between stromal CD10 expression and lymph node metastasis (p-value = 0.232), which is similar to the findings of some studies [5,7,8].

Among the 50 cases of invasive breast carcinoma, we observed a statistically significant correlation of CD10 in relation to ER-negative and PR-negative status with a p-value of 0.045 and a p-value of 0.048, respectively. The p-value was 0.119, which shows a statistically insignificant association between stromal CD10 expression and HER2-neu status. Studies conducted by a few authors [5-8] showed a positive correlation between CD10 expression by stromal cells and PR- and ER-negative status, which is in concordance with the present study.

The role the CD10 marker plays in drug development is another significant function. It is no longer necessary to create therapies that target the cancer epithelial cells when treating breast cancer; instead, it is now possible to create medications with improved delivery systems, maximum efficacy, minimal toxicity, and the ability to alter the tumor microenvironment/stroma [6].

Due to this, peptide prodrugs that can be broken down by peptidases found in the tumor environment have been developed, improving their maximal efficacy with the least amount of side effects. CD10 can cleave similar peptide prodrugs like “N-succinyl-alanyl-L-isoleucyl-L-alanyl-L-leucyl-Dox (sAIAL-Dox)” and “CPI-0004Na” [6,9]. With more potency than Dox alone, this “proteolytic cleavage” produces “leucyl-Dox” which can penetrate cells and produce intracellular Dox [3]. The recognized inhibitor of CD10 enzymatic activity, phosphoramidon, reduces the cytotoxicity of CPI0004Na. Consequently, routine CD10 staining may aid in determining the course of treatment for breast carcinoma cases [6].

In the present study, we correlated stromal CD10 expression in breast carcinoma and found a strong association of stromal CD10 with negative prognostic factors such as higher tumor grade, ER negativity, and PR-negative status signifying its association with aggressive behavior of the tumor. Hence, it can be considered an independent prognostic marker for breast carcinoma.

The limitations of the study were: small sample size, some variations may be there in the CD10 expression by stromal cells due to heterogeneity of the breast carcinoma as immunohistochemistry was performed on one representative section of the tumor. HER2 evaluation in equivocal cases was performed only by immunohistochemistry as fluorescence in situ hybridization (FISH) was not available.

## Conclusions

The expression of CD10 by stromal cells was strongly associated with negative prognostic factors such as higher tumor grades, ER negativity, and PR-negative status. CD10 could serve as a potential target for the creation of novel drugs, but further functional studies are necessary to clarify the signaling mechanisms that lead to its over-expression in the stroma of invasive breast carcinoma.
